# Calcium Triggered L_α_-H_2_ Phase Transition Monitored by Combined Rapid Mixing and Time-Resolved Synchrotron SAXS

**DOI:** 10.1371/journal.pone.0002072

**Published:** 2008-04-30

**Authors:** Anan Yaghmur, Peter Laggner, Barbara Sartori, Michael Rappolt

**Affiliations:** Institute of Biophysics and Nanosystems Research (IBN), Austrian Academy of Sciences, Graz, Austria; Massachusetts Institute of Technology, United States of America

## Abstract

**Background:**

Awad et al. [1] reported on the Ca^2+^-induced transitions of dioleoyl-phosphatidylglycerol (DOPG)/monoolein (MO) vesicles to bicontinuous cubic phases at equilibrium conditions. In the present study, the combination of rapid mixing and time-resolved synchrotron small-angle X-ray scattering (SAXS) was applied for the in-situ investigations of fast structural transitions of diluted DOPG/MO vesicles into well-ordered nanostructures by the addition of low concentrated Ca^2+^ solutions.

**Methodology/Principal Findings:**

Under static conditions and the in absence of the divalent cations, the DOPG/MO system forms large vesicles composed of weakly correlated bilayers with a *d*-spacing of ∼140 Å (L_α_-phase). The utilization of a stopped-flow apparatus allowed mixing these DOPG/MO vesicles with a solution of Ca^2+^ ions within 10 milliseconds (ms). In such a way the dynamics of negatively charged PG to divalent cation interactions, and the kinetics of the induced structural transitions were studied. Ca^2+^ ions have a very strong impact on the lipidic nanostructures. Intriguingly, already at low salt concentrations (DOPG/Ca^2+^>2), Ca^2+^ ions trigger the transformation from bilayers to monolayer nanotubes (inverted hexagonal phase, H_2_). Our results reveal that a binding ratio of 1 Ca^2+^ per 8 DOPG is sufficient for the formation of the H_2_ phase. At 50°C a direct transition from the vesicles to the H_2_ phase was observed, whereas at ambient temperature (20°C) a short lived intermediate phase (possibly the cubic Pn3m phase) coexisting with the H_2_ phase was detected.

**Conclusions/Significance:**

The strong binding of the divalent cations to the negatively charged DOPG molecules enhances the negative spontaneous curvature of the monolayers and causes a rapid collapsing of the vesicles. The rapid loss of the bilayer stability and the reorganization of the lipid molecules within ms support the argument that the transition mechanism is based on a *leaky* fusion of the vesicles.

## Introduction

Self-assembled nanostructures play an important role in cell life. Over the last decades, learning from the self-assembly of molecular building blocks in nature and mimicking especially the architecture of biomembranes by model membrane systems have lead to various applications such as composite materials synthesis, novel optics and catalysts, as well as to the development of novel functional food, cosmetics, and drug nanoparticulate carriers [2]–[7]. Therefore, it is important to study their formation processes and their stability under various physicochemical conditions such as varying temperature, pressure, or the addition of solutes (surfactant, cosurfactant, hydrophilic or hydrophobic additives, salts, etc.) [8]–[12]. Moreover, understanding biological relevant functions and controlling efficiently processes on a mesoscopic level is desirable. Thus, research on the mechanisms of structural transformations in self-assembled systems and the detection of possible intermediate phases has aroused great interest [8]–[13]. We note that under realistic circumstances, the structural transitions taking place in cell life or any model system are often different from those observed under equilibrium conditions [9], [10], [12], [13].

Combining rapid mixing and X-ray scattering techniques has become an important tool for investigating the dynamics of structural transitions in the self-assembled systems. Recent reviews [9], [10], [13] showed the power of integrating stopped-flow devices in synchrotron small-angle X-ray (SAXS) or neutron scattering (SANS) beamlines. It allows in-situ millisecond time-resolved experiments. There is also a growing number of investigations devoted to elucidating the morphological transitions in amphiphilic systems [8]–[14]. For instance, Weiss et al. [8] reported on the spontaneous formation of unilamellar vesicles (ULVs) by rapidly mixing two oppositely charged micellar solutions. In other investigations, the internal transfer of materials from nanostructured emulsions [15], the impact of rapid mixing of salt solutions with phospholipid vesicles [16], [17], and the salt-induced formation of calcium carbonate [18] have been studied by time-resolved SAXS. Coupling rapid mixing to other techniques helps also to unravel other interesting processes [19], [20]. For instance, the folding pathway of an α-helical membrane protein in lipid vesicles has been kinetically studied by using fluorescence and absorption spectroscopy [19]. Another example is combining Fourier transform infrared spectroscopy with SAXS for monitoring the rapid hydrolysis and the condensation of metal alkoxides [20].

In this work, we applied the stopped-flow method in combination with synchrotron SAXS to study the impact of Ca^2+^ on an anionic bilayer model system. In nature, the associations of divalent cations to negatively charged phospholipids have a very important role in several processes [21]–[23]. For instance, the strong binding of Ca^2+^ ions to anionic phospholipids in biological membranes is crucial for several functions such as in fusion processes, the protein regulation, the transportation of molecules across the membranes, and the neural signal transduction [21]–[31].

It is a difficult task though to fully understand the role of Ca^2+^ in the biological processes due to their complexity. The strong affinity of this divalent cation to anionic lipids leads to the formation of coordination complexes with one or more of lipid phosphate groups and thus causes drastic changes in the phase behavior of the biomembrane. Therefore, a great share of research studies is dedicated to understanding the impact of Ca^2+^ ions on phospholipid model systems, which are mimicking the structure of biologically relevant membranes in a simple manner [21]–[29], [32]–[35]. In particular, Ca^2+^-induced lamellar to non-lamellar structural transitions in anionic phospholipid systems have been repeatedly studied. Theses investigations were in great part also motivated by the significant role of these transitions in fusion driven processes like in endo- and exocytosis [24], [27], [35]–[39]. In this context, the influence of Ca^2+^ on anionic cardiolipin (CL) vesicles is remarkable. Already the addition of a low concentration of divalent cations induces the transition from the fluid lamellar (L_α_) to the inverted type hexagonal phase (H_2_) [24], [27], [35], [38], [39]. A similar behavior was also observed for membranes based on phosphatidic acid (PA) [36], [37].

Nevertheless, membrane curvature is not only driven by divalent salt ions, but some special peptides and proteins may also induce the formation of non-lamellar structures [40]–[44]. Recently, we found that the addition of short charged designer peptide surfactants can be used to stabilize different non-lamellar mesophases [44]. However, the growing interest to study also non-lamellar structures of other surfactant-like molecules is mainly stimulated by their biological relevance [45]–[52]. Moreover, the non-lamellar phases (such as the inverted types of hexagonal and cubic phases) are not only characterized in the bulk non-dispersed state, but their fragmentation into kinetically stabilized submicron sized dispersed particles have also received considerable attention [45], [46], [53]–[67].

**Figure 1:**
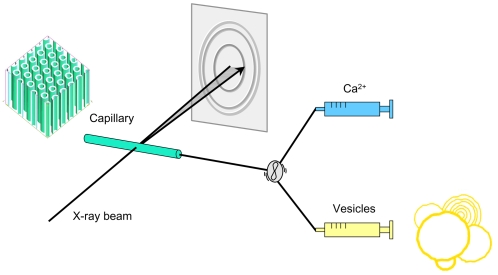
Schematic of the combined stopped-flow and synchrotron SAXS set-up. In the stopped-flow apparatus, one syringe contained a buffer with Ca^2+^ ions, whereas the other contained DOPG/MO-based vesicles. The rapid mixing was conducted within 10 ms and the formation of the inverse hexagonal phase (H_2_) was followed by millisecond time-resolved SAXS.

In this study, our main objective focuses on the dynamical behavior of DOPG/MO-based vesicles after rapid mixing with low concentrated Ca^2+^ solutions. Awad et al. [1] reported on the transition of multi- (MLV) and unilamellar (ULV) DOPG/MO-based vesicles into bicontinuous cubic phases at certain Ca^2+^ concentrations. In their work, SAXS experiments were carried out under static conditions. Our rapid-mixing experiments on the same lipid system can be considered complementary to those done in [1]. Our main interest focuses on the dynamic salt-lipid interactions. The applied set-up is schematically illustrated in **Figure 1**. Another major goal is to gain insight into the mechanism of calcium-triggered structural transition pathways in binary lipid systems, i.e. to elucidate the Ca^2+^ induced vesicle-vesicle interactions, to detect possible formation of intermediate phases, and possibly to learn how to control the overall process.

## Results and Discussion

### 1. Characterization of DOPG/MO-based Vesicles

**Figure 2:**
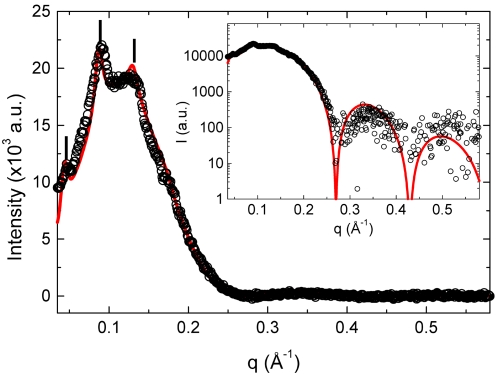
Background subtracted SAXS pattern of DOPG/MO-based vesicles at 25°C in the absence of Ca^2+^ ions. The investigated vesicles are composed of DOPG and MO at a molar ratio of 30∶70 with a total lipid content of 7 wt%. The best global fit to the experimental data is given by a solid red line (cp. data analysis). The inset shows the quality of the fit at higher *q*-values. The structural bilayer parameters are summarized in Table 1.

To characterize the DOPG/MO-based vesicles with a molar ratio of 30∶70 long static exposures were taken at 20, 25 and 50°C, respectively. Under static conditions, the vesicles are stable for at least several weeks. In addition, there was no significant change in the overall properties of the vesicular dispersions at the temperature range of 20–50°C. A typical SAXS pattern at 25°C after subtraction of the background arising from water and sample holder is displayed in **Figure 2**. The weak membrane correlations can already be judged from the low intensity of the first three diffraction peaks. In other words, the pattern is mainly dominated diffuse scattering.

**Table 1:** Fit parameters derived from the GAP evaluation of the SAXS pattern (Figure 2) for the DOPG/MO-based vesicles (7 wt% lipid, T = 50°C).

Parameter	Value
*d* (Å)	144.0±0.2
*N _mean_*	2±1
*η*	0.091±0.004
*z_H_* (Å)	18.3±0.3
*σ_H_* (Å)	2.95±0.2
*σ_C_* (Å)	7.40±0.3
*ρ_R_*	−1.00±0.06
*N _diff_*	0.94±0.39

**Figure 3:**
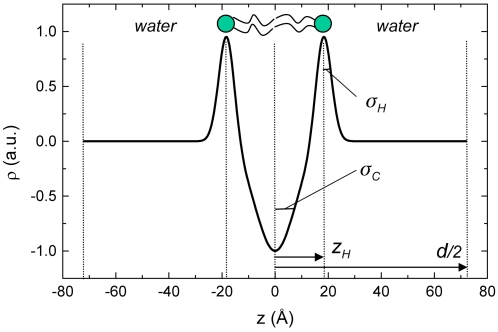
Electron density bilayer model of the DOPG/MO vesicles at 25°C in the absence of Ca^2+^ ions. This model is composed of one Gaussian representing the electron density distribution of the polar headgroups at ±*z_H_* and a second for the hydrophobic core with its centre at the bilayer mid-plane. The corresponding widths of the Gaussians are given by the standard deviations *σ_H_* and *σ_C_*, respectively. *d* defines the lattice spacing of the L_α_ phase. The obtained results refer to the data of Figure 2.

The *d*-spacing, the headgroup position, *z_H_*, the headgroup width, *σ_H_*, and the width of the hydrophobic core, *σ_C_*, are defined in **Figure 3**. *ρ_R_*, is the relative electron density of the bilayer trough set in relation to the headgroup density, *N_mean_*, the membrane correlation number, *N_diff_*, the fraction of diffuse scattering and *η*, the Caillé parameter.

The applied global model for fitting SAXS patterns of fluid lamellar phases, which is described in Materials and Methods, shows good agreement with the measured intensity over the whole measured *q*-range. In the low *q*-regime, the first diffuse maximum is displayed together with the Bragg peaks and the fit quality of the high *q*-regime can be judged from the log/lin plot in the inset of **Figure 2**. The derived parameters of the global fitting method are summarized in **Table 1**, and the obtained bilayer model is illustrated in **Figure 3**. Our results reveal that the interbilayer distance in these weakly ordered bilayer stacks of the DOPG/MO vesicles is *d* = 144 Å, which is relatively big as compared to neutral phospholipid/water systems [48]. This is caused by electrostatic repulsion between the charged DOPG/MO membranes. Hence, the L_α_ system is highly swollen indicating further that the overall vesicle size must be relatively large as compared to neutral lipid MLVs. Moreover, the big interlamellar distance explains also the low membrane correlation number, *N_mean_* (**Table 1**). The highest scattering contribution is given by the diffuse scattering (*N_diff_* = 94%). It arises from positionally uncorrelated membranes that do not “see” each other. This can be attributed either to the occurrence of defects, or due to the membrane undulations. It could be also simply related to the coexistence of ULVs. The measured bilayer properties are typical for the fluid L_α_ phase. The determined bilayer thickness (2*·z_H_* = 36.6 Å, **Figure 2**) agrees also well with the previously reported value for the binary MO/water system [59]. The mean square fluctuations in the water spacing between bilayers - expressed in the Caillé parameter, *η* - are similar to those of other weakly bound fluid membrane systems [68].

### 2. In-situ Monitoring of Direct L_α_-H_2_ Transition

**Figure 4:**
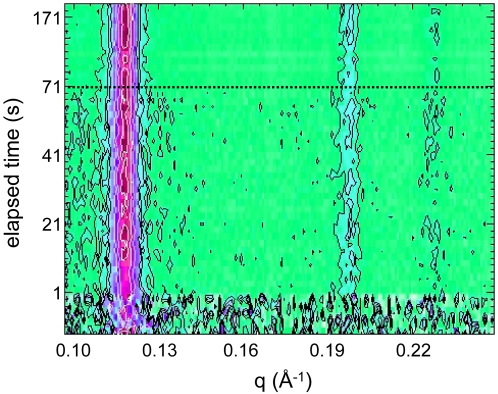
Time-resolved X-ray pattern of the rapid calcium-triggered H_2_ phase formation at 50°C. The vesicle dispersion contained DOPG/MO with a molar ratio 30∶70 (7 wt% lipid), and the final salt concentration was 20 mM. The contour plot clearly displays the first three reflections of the H_2_ phase; no indication for the formation of an intermediate phase is spotted.

**Figure 4** shows an example of the calcium induced structural L_α_-H_2_ phase transitions observed in DOPG/MO-based large vesicles at 50°C (30∶70 mol/mol with a total lipid content of ∼7 wt %). The CaCl_2_ concentration after rapid mixing was 20 mM. The real-time evolution of the SAXS patterns demonstrates the drastic impact of Ca^2+^ ions on tuning the curvature of DOPG/MO monolayers. Intriguingly, there is a very fast and direct transition of the vesicles to the H_2_ phase. The three characteristic peaks of the H_2_ phase, which are identified by the (10), (11), and (20) reflections, are clearly observed. This observation indicates that the vesicles lose their stability immediately after the rapid-mixing procedure and the structural transition occurs during the first milliseconds of our investigation. Herein, we do not have any indication for the formation of an intermediate structure.

The driving force for the fast L_α_-H_2_ phase transition is the strong electrostatic interactions of Ca^2+^ ions with the phosphate group of the anionic DOPG lipid. The divalent cation is screening the repulsive forces between the negative charges of DOPG molecules in the membrane, and its strong binding leads to the Ca^2+^-DOPG complex formations. This strong ion binding was reported for various phospholipid systems [21], [32]–[34], [69]–[72]. Lehrmann and Seelig [72] suggested a two-step mechanism for describing the complex of negatively charged phospholipid-Ca^2+^ phases. Firstly, the divalent ions are bound tightly to the anionic groups of the lipid molecules and thus leading to an increase in the local Ca^2+^ concentration at the lipid/water interface. Secondly, the strong direct interactions of Ca^2+^ ions with the phosphate group induce the formation of coordination complexes, in which an ion is bound via the phosphate group to one or more lipid molecules. In another article, Böckmann and Grubmüller [73] showed in simulation studies for neutral and zwitterionic phospholipid bilayers that the coordination of Ca^2+^ with lipid bilayers is a multistep process involving the appearance of a sequence of intermediates, when the ions are bound to the carbonyl group of the phospholipid. Furthermore, Pedersen et al. [70] reported recently that Ca^2+^ ions move rapidly within nanoseconds from the bulk phase of water to the lipid bilayers and are localized in a narrow (∼10 Å) band near the phosphate group. This differs significantly from the behavior of monovalent ions such as Na^+^ [71], [73]–[75].

In a first attempt to explain the impact of adding Ca^2+^ ions on the DOPG/MO-based vesicles, we follow the simple approach describing the ‘effective’ molecular geometry of the involved lipids and its influence on the formation of diverse self-assembled nanostructures. It is expressed as the critical packing parameter (CPP) or the molecular shape factor, which is defined as *v_s_*/*a_0_l*, where *v_s_* is the hydrophobic chain volume, *a_0_* is the headgroup area, and *l* is the hydrophobic chain length [76]. In literature, it was shown that there is a strict dependence of the CPP on different parameters such as the individual surfactant's shape, temperature, hydration, the presence of hydrophilic or hydrophobic guest molecules, and electrostatic forces [1], [36], [44], [53], [63], [77].

To better understanding the behavior of studied DOPG/MO system in the presence of divalent cations, one should recall that the fully hydrated binary MO/water system displays at ambient temperatures the bicontinuous cubic phase with Pn3m symmetry. The successive addition of sufficient DOPG molecules leads to the formation of the L_α_ phase [1]. This structural transition is attributed to the electrostatic repulsions between the negative charges of the DOPG molecules incorporated into the electrically neutral MO-based membrane. This means an increase of the distance of the negative charges and an increase in the *a_0_* value at the water/lipid interface. Hence, the electrostatic repulsions are sufficient to significantly decrease the CPP value and to destabilize the former bicontinuous cubic phase [1]. It was further demonstrated that this structural transition does not occur in the presence of 1.0 M NaCl indicating that the electrostatic forces are playing a crucial role in controlling the nanostructure [1]. The individual shape of DOPG molecules, which can be considered as rod-like, works on the CPP value in the same direction, i.e. inducing a less negative spontaneous curvature. This is also confirmed well by other investigations, in which it was proved that mixing a surfactant favoring the formation of lamellar phases (such as DOPG) with a lipid favoring the formation of non-lamellar phases (such as MO) induces non-lamellar to lamellar phase transitions [53], [78]–[80].

Returning to our studied system, we found that the addition of Ca^2+^ ions to the negatively charged DOPG/MO vesicles has a very strong impact on the lipidic nanostructure. Ca^2+^/DOPG complexes decrease the electrostatic repulsions between neighbouring DOPG molecules and hence, cause a decrease in the *a_0_* value. Intriguingly, our results show that already at relatively low salt concentrations (DOPG/Ca^2+^ ratios>2.0, confer **Table 2**), the CPP value increases so drastically that a direct L_α_- H_2_ structural transition takes place, i.e. without forming any longer living intermediate bicontinuous cubic phase. For negatively charged single phospholipid bilayers, it is well known that the strong binding of Ca^2+^ ions to the phosphate group such as in phosphatidylglycerols (PGs) and phosphatidlyserines (PSs) is entropy driven and causes a dehydration of the membrane (a decrease in *a_0_*). Moreover, a *condensing effect* reflected by a tighter packing of the fatty acyl chains is observed [32]–[34], [70], [81]–[83].

**Table 2:** The molar ratios of DOPG/[Ca^2+^] and (DOPG+MO)/[Ca^2+^] after rapidly mixing the DOPG/MO-based vesicles with PIPES buffer (pH 7.0) containing 68 mM Ca^2+^ ions.

Exp. No.	Volume A (%)	Volume B (%)	Final Ca^2+^ conc. (mM)	DOPG/[Ca^2+^]	(DOPG+MO)/[Ca^2+^]
1	10	90	61	0.1	0.2
2	30	70	48	0.3	0.9
3	50	50	34	0.6	2.1
4	70	30	20	1.5	4.9
5	90	10	6.8	5.5	18.4
6	93	7	4.8	8.3	27.8
7	94	6	4.1	9.9	32.8
8	95	5	3.4	12.0	40.0

The investigated vesicles in the absence of Ca^2+^ ions were composed of DOPG and MO at a constant molar ratio of 30∶70 and the total lipid content was 7 wt%. Eight different rapid-mixing investigations with different volume ratios of the vesicles (volume A) to the buffer (volume B) are summarized. In addition, the final Ca^2+^ concentrations are given.

**Figure 5:**
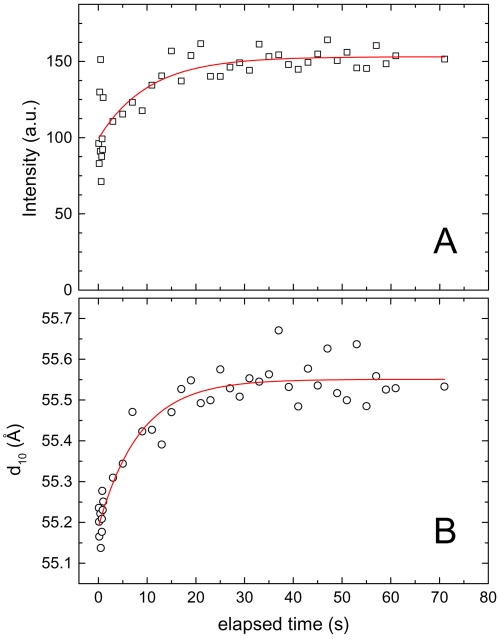
Time dependence of the intensity (A), and the *d*-spacing (B) of the first order reflection of the rapidly formed H_2_ phase referring to the experiment given in Figure 4. The solid red line shows the best single exponential fit to the data. The time constant, *k*, was determined to be 0.10±0.03 and 0.12±0.02 sec^−1^ for panels (A) and (B), respectively.

**Figure 5** displays the time evolution of the intensity of the first order diffraction peak (panel A), and its corresponding *d*-spacing (panel B) of the H_2_ phase, which is formed directly after the rapid-mixing process. A simple single exponential function is used to fit the data and it is represented by a full red line. Both, the intensity and the lattice spacing reflect the same kinetics (*k* = 0.10±0.03 and 0.12±0.02 sec^−1^, respectively). The full formation of the H_2_ phase is accomplished after ∼20 sec. However already 100 ms after the rapid mixing, ∼50% of the material is ordered in the form of aggregated inverted lipid nanotubes (panel A), and the restructuring step thereafter is minor as can be seen in the small lattice changes (∼0.5 Å) (panel B). Thus, at the given time-resolution of 100 ms per frame, the early steps of the transition are not detected: the salt-binding (nanosecond scale), the adhesion of the membranes, and finally the initial part of the conversion to the H_2_ phase are obscured. In this regard, we can only argue that these first steps are rapid (nano- to millisecond time scale), and most probably the transformation to non-lamellar structure takes place directly. The good agreement of the single exponential function with the experimental data supports our arguments that any hypothetical formation of an intermediate phase has at most a lifetime of few milliseconds.

**Figure 6:**
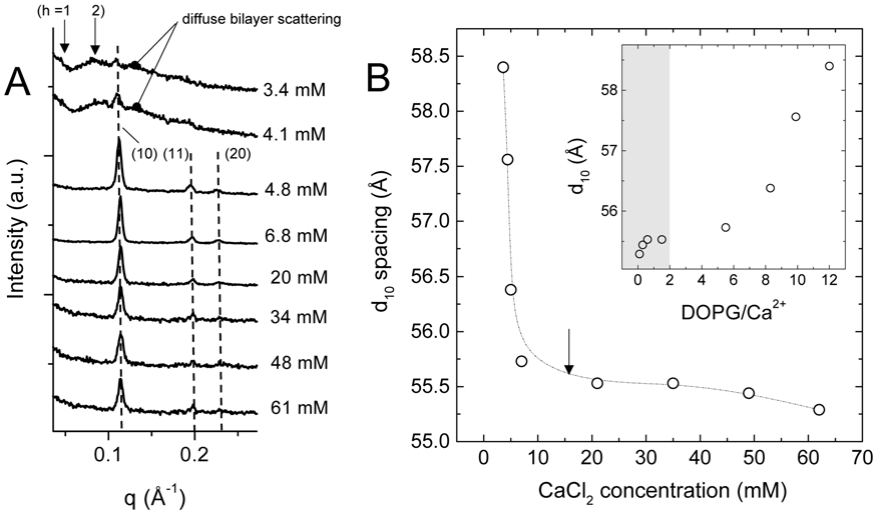
Comparison of the calcium-induced H_2_ phase in dependence of the final salt concentration. All rapid-mixing experiments were carried out at 50°C. (A) The SAXS patterns of the DOPG/MO-based aqueous dispersions are displayed 71 s after the rapid mixing, i.e. approximately one minute after the turnovers were completed (Figure 5). It should be pointed out that for the two lowest salt concentrations the H_2_ phase is coexisting with weakly correlated bilayers (the positions of the very weak first two diffraction orders are marked by arrows). (B) The *d_10_*-spacing of the H_2_ phase is displayed as a function of the final Ca^2+^ concentration. The inset shows the *d*-spacing in dependence of the DOPG/Ca^2+^ ratio. The electroneutral regime is highlighted in light grey.

In order to study the effect of different Ca^2+^ concentrations on the DOPG/MO membranes, we carried out a series of rapid-mixing experiments. **Figure 6a** presents the SAXS scattering curves for the dispersions containing various amounts of Ca^2+^ ions 71 sec after rapid mixing. Interestingly, already at very low Ca^2+^ concentrations the strong binding of Ca^2+^ to the polar interface induces the formation of the H_2_ phase. At 3.4 mM, which is the lowest divalent ion concentration applied, the typical SAXS pattern of DOPG/MO vesicles is accompanied by the appearance of a small peak at *q* value of approx. 0.107 Å^−1^. It indicates a biphasic dispersion consisting of H_2_ traces coexisting with vesicles. The observed peak refers to the (10) reflection of a newly formed H_2_ phase. With a further increase of Ca^2+^ ions concentration, the observed peak's intensity and sharpness increase until having a full transformation to the H_2_ phase (at 4.8 mM). In **Figure 6b**, the *d_10_*-spacing value of the H_2_ phase is plotted versus Ca^2+^ ions concentration. In the regime of low Ca^2+^ concentrations (<10 mM), there is a sharp decrease of approx. 3 Å in the *d_10_*-spacing value with increasing Ca^2+^ concentration. The results reveal also that a further increase of the Ca^2+^ concentrations (>10 mM) has insignificant influence on the structure parameter. A plausible explanation is associated with the compensation of the negatively charged DOPG headgroups by Ca^2+^ ions. Arseneault and Lafleur [84] investigated the binding of Ca^2+^ ions with the binary 1-palmitoyl-2-oleoyl-*sn*-glycero-3-phosphoglycerol (POPG)/water system by using isothermal titration calorimetry. They found that the binding saturation of Ca^2+^ ions is completed when the electroneutrality is reached, in which the salt-binding site in the membrane consists of 2 PGs and 1 Ca^2+^. This conclusion was also drawn from the analysis of Ca^2+^ binding to PS bilayers [85], and it is supported by similar binding ratios reported for pure PG bilayers [82]. The key role of electroneutrality is confirmed also by our data: as long as electroneutrality is not reached, major rearrangements take place in the H_2_ phase. Conversely, for ratios of DOPG/Ca^2+^ less than 2 no significant lattice changes are observed (see inset of **Figure 6b** and **Table 2**).

As mentioned in the introduction, Ca^2+^ ions induce also the L_α_-H_2_ phase transition in the CL-based membranes [24], [27], [35] and in the PA-based systems [36], [37]. To summarize this part of our results, it must be stressed firstly that the local Ca^2+^ concentration is the main driving force for inducing membrane curvature, and secondly, the availability of salt at the membrane interfaces in the rapid-mixing experiments is almost immediate (<100 ms). This means that the vesicle-vesicle interactions are probably highly leaky. Rapid collapsing of vesicles with early loss of their bilayer structure favors the formation of the H_2_ phase. Leaky vesicle-vesicle interactions have also been suggested for the H_2_ phase formations induced by the divalent cations Sr^2+^ and Ba^2+^ [24].

### 3. Detection of an Intermediate Phase at Ambient Temperature

**Figure 7:**
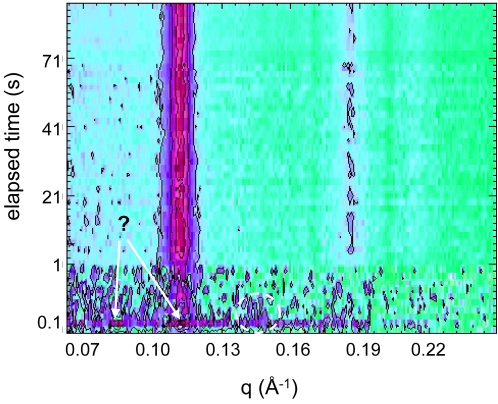
Time-resolved X-ray pattern of the calcium induced H_2_ phase formation at 20°C. The vesicle dispersion contained DOPG/MO with a molar ratio 30∶70 (7 wt% lipid), and the final salt concentration was 34 mM. The contour plot displays the first three reflections of the H_2_ phase, but its formation is not immediate. In the first 400 ms, an intermediate phase is apparent. Two strong reflections are indicated by arrows and one weaker peak is circled by a dashed line.

**Figure 8:**
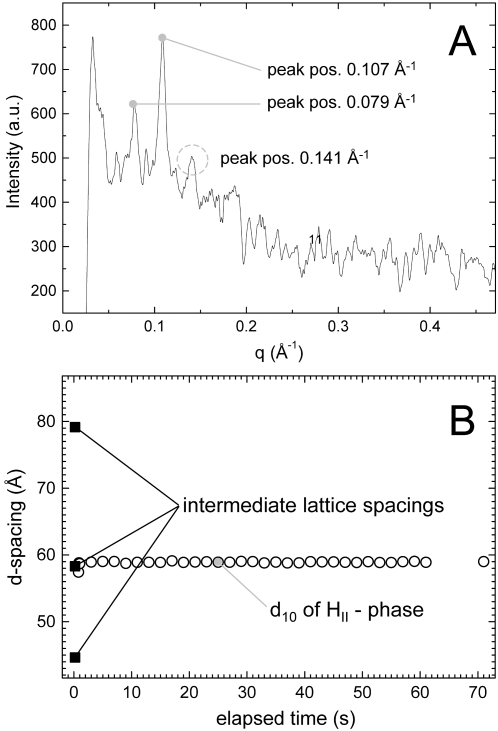
Intermediate formation referring to the rapid-mixing experiment of Figure 7. (A) The SAXS pattern is averaged from the data taken in the range of 100–400 ms after the rapid mixing. It indicates a possible formation of bicontinuous cubic phase of the symmetry Pn3m. The observed Bragg peaks and their presented *q*-values, suggest a coexistence with the H_2_ phase (for details see text). (B) Temporal evolution of the observed lattice spacings.

The association of divalent cations to negatively charged phospholipid membranes is not only affected significantly by the salt concentration, but it is also enhanced by varying temperature. Thus, we repeated the same rapid-mixing experiments as done in the previous section (**Figure 4**), but now at an ambient temperature (20°C). **Figure 7** shows a series of SAXS patterns taken during this investigation. The CaCl_2_ concentration after mixing was 34 mM. The two most striking features are the appearance of a possible intermediate bilayer nanostructure with a short life time (seen 100–400 ms after mixing) and the rapid induction of the H_2_ phase. The two strongest reflections of the intermediate phase are marked by arrows and an additional weaker reflection is circled with a dashed line. A closer look on the diffraction pattern in this time window is given in **Figure 8a**. The *q*-values of the three observed peaks are 0.079, 0.107, and 0.141 Å^−1^, respectively. It should be pointed out that the diffuse background is typical for bilayer-based aggregates (compare **Figure 2**) and definitely is too high to origin only from the appearance of the H_2_ phase. Unfortunately, the assignment of this intermediate phase is not unambiguous due to the low signal to noise ratio in the SAXS scattering curve. However, it is easy to realize that the second peak, which is developing with time is most probably to be identified as the (10) reflection of the H_2_ phase (**Figure 8b**). It can be clearly seen, when the intensity of this reflection has reached its maximum, that also the characteristic (11) and (20) reflections are observed (**Figure 7**). We note that under equilibrium conditions, a stepwise increase of the Ca^2+^ concentration in the DOPG/MO system leads to the structural transformation from the L_α_ to the cubic Im3m and Pn3m phases, respectively [1]. On this background, the appearance of the additional peaks at 0.079 and 0.141 Å^−1^ could indicate the formation of an intermediate bicontinuous cubic structure coexisting with the H_2_ phase. These peaks are compatible with the reflections (110) and (211) in bicontinuous cubic phase with Pn3m symmetry and a lattice parameter of 112 Å, which agrees well to the reported values of the Ca^2+^-induced Pn3m phase investigated in [1]. The (111) and (200) reflections of the proposed Pn3m phase are not detected, most probably due to the low signal to noise ratio and possible overlap with the (10) reflection of the coexisting H_2_ phase. Alternatively, the intermediate formation of the Im3m phase is unlikely, since the lattice parameters should fall into a range of 150–180 Å [1], and a coexisting L_α_ phase could not explain the weak peak at 0.141 Å^−1^ (for this hypothesis, the 2^nd^ order peak should be spotted at 0.158 Å^−1^). In summary, it is most likely that **Figure 8a** shows an intermediate phase of bicontinuous cubic phase of the symmetry Pn3m coexisting with a newly formed H_2_ phase. It is also remarkable that the complete H_2_ phase formation occurs very fast as soon as the intermediate phase has vanished. De Kruijff et al. [38] reported also on the possible occurrence of an intermediate cubic phase in a Ca^2+^-induced CL bilayer to H_2_ phase transition.

Our findings indicate that the impact of Ca^2+^ ions on the spontaneous monolayer curvature is higher at 50°C, since for the given time-resolution of 100 ms no intermediate phase could be detected during the L_α_-H_2_ phase transition. For this behavior, two main reasons can be identified. First, at higher temperatures the chain pressure increases, which decreases the value of the spontaneous monolayer curvature, and hence favors the formation of the H_2_ phase. Second, increasing temperature enhances also the dehydration of the hydrophilic headgroups. Especially, the strong dehydration of the MO headgroups leads to a further decrease in the monolayer curvature. Therefore, as shown in **Figures 4** & **8b**, also the *d_10_*-spacing of the fully formed H_2_ phase decreases by approx. 3.5 Å with increasing temperature from 20 to 50°C. A similar temperature behavior is also known for the fully hydrated pure MO-based system [86], [87].

### 4. The Importance of the Experimental Protocol – Sample History

It is very interesting that our results presented in the previous sections are different from those published under static conditions by Yamazaki and his coworkers [1]. One crucial difference is given by the sample preparation protocol. In [1], the aqueous dispersions were centrifuged for half an hour at 13000 g and thereafter the excess of water was removed. This means that the SAXS investigations were carried out on so called ‘pellets’. Normally, one would not expect that such a treatment could influence the nanostructure, because the salt concentration should remain the same and furthermore, the full hydration conditions should still be maintained. In this regard, our SAXS measurements on pellets confirmed the results of [1]: at 20.4 mM Ca^2+^ concentration, the Im3m phase forms, while at a higher concentration (34.0 mM) the Pn3m phase is observed (data not shown). Intriguingly, in our study keeping all experimental conditions identical, but refraining from the removal of excess of water, and thus, measuring a *real* DOPG/MO-based aqueous dispersion, leads to the formation of the Ca^2+^-induced H_2_ phase at both salt concentrations. In order to check the stability of the formed H_2_ phase, we also remeasured the same dispersions after 1 and 2 weeks: the H_2_ phase remained stable. This finding at least confirms the outcome of our time-resolved experiments carried out with vesicles coexisting with excess water.

However, it remains the question why Ca^2+^ ions induce in the pellet system bicontinuous cubic phases with moderate membrane curvatures rather than the H_2_ phase. Although any tentative to answer this question remains highly speculative, there are in principle only two possible explanations: either the membranes in the pellets display a lower salt affinity or for some reason the DOPG/MO ratio gets altered in favor of MO during the last steps of pellet production. The first scenario seems somehow awkward, because temperature and salt concentration are kept the same, but also in other studies the influence of the sample morphology on the salt affinity was stated. For instance, Arsenault and Lafleur [84] demonstrated especially for small ULVs with stressed bilayer curvatures that the salt affinity is altered. In our case, either the centrifugation step or the fact that the pellets resemble rather a pure liquid crystalline phase rather than a ‘real’ aqueous dispersion might have lowered the membrane affinity for the salt. Thus, with an overall decrease in the salt-binding affinity, higher salt concentrations would be needed to induce the H_2_ phase. Secondly, it could be that the centrifugation of the DOPG/MO-based dispersions for forming pellets induces the segregation of a portion of DOPG molecules, which results in the formation of DOPG-rich vesicles associated with Ca^2+^ ions in the excess water. This effect would suggest that excluding part of DOPG molecules from the DOPG/MO membrane favors the formation of bilayers. In other words, a more MO-rich system would explain the formation of the cubic phases. In this context, it is also noteworthy from the previous studies that Ca^2+^ ions mixed with DOPG/MO ULVs induced fragments of the sample to be assembled in the cubic Pn3m phase, which accumulated at the walls of the sample container [1]. In this particular case, it could also be that a phase separation occurred, i.e. leading to relatively MO-rich fragments coexisting with DOPG-rich vesicles in excess of water. This viewpoint is supported by the known fact that the association of Ca^2+^ with membranes composed of different lipids can enhance the phase separation into microdomains [72]. In this regard, it is also possible to think about a combined effect: removing unwillingly part of the DOPG material with the excess of water, means a simultaneous reduction in salt concentration due to the strong association of Ca^2+^ to DOPG molecules. This of course would also lower the spontaneous monolayer curvature.

### 5. Mechanism of the Lamellar (L_α_) to the Bicontinuous Cubic (Q_2_) and to the Hexagonal (H_2_) Phase transitions

Owing to the negatively charged surfaces of the DOPG/MO bilayers, neighboring membranes are only weakly correlated (**Figures 2** and **3**). However, as soon as the vesicle systems are exposed to the Ca^2+^ solution, strong complexes between the DOPG headgroups and the Ca^2+^ ions are formed. Consequently, the electrostatic repulsion vanishes and the bilayers approach each other. The monitored fast structural transition is attributed to the effective vesicle-vesicle interactions between different vesicles or between different lamellae within one vesicle. For pure PG/water systems, it was even proposed [82], [83] that the strong association of divalent cations with PGs leads to the formation of highly ordered dehydrated fluid lamellar phases, in which the divalent cations act as a bridge between planar bilayers. Thus, the first step of vesicle-vesicle interactions is believed to involve a rapid collapsing of the vesicles. The second important salt effect concerns the monolayer curvature. As discussed above, the integration of the divalent cations into the polar interface results in higher negative spontaneous curvatures, and finally promotes the formation of non-lamellar phases.

**Figure 9:**
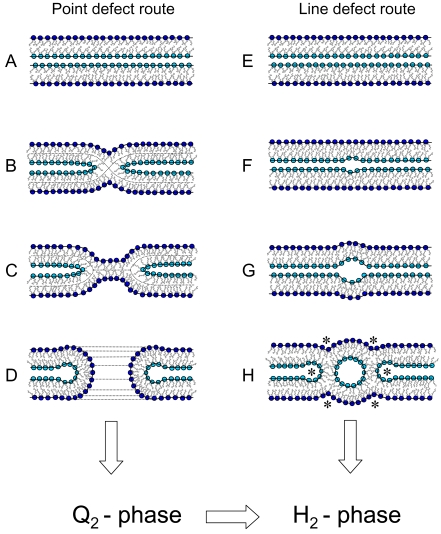
Two schematics of the proposed pathways from the bilayer to the inverted monolayer tube transition. On the left hand side, the classical vesicle fusion route is depicted. The formation of pores is widely believed to be the prerequisite for the formation of bicontinuous cubic (Q_2_) nanostructures [87], which upon further curvature frustration may transform into self-assembled monolayer tubes (H_2_ phase). On the right hand side, the direct formation of an inverse lipid nanotube between two opposed bilayers is illustrated. For better understanding of the structural conversions, the headgroups of opposed monolayers are shown in light blue whereas the rest are depicted in blue (for further details see text).

In **Figure 9**, two possible transition routes are depicted. They are generally different, but both can lead to the final formation of the H_2_ phase. The first pathway describes the classical fusion steps of two opposed membranes and bases on the formation of *point defects* [88]: after two vesicles adhere (A), an intermembrane attachment site can form (B, stalk-like topology), which then converts into an extended area of intermembrane contact (C, called transmonolayer contact or hemifusion) from which a pore evolves (D). Finally, the creation of numerous pores within an interconnected vesicular system is believed to be able to transform into Q_2_ phases [89]. The transformation from Q_2_ to H_2_ phases is not well understood on a molecular level, but it has been experimentally proven that this transition takes place in various lipid/water systems [87], [90]. In contrast, the second mechanism focuses on describing the possible pathways for the direct L_α_-H_2_ structural transition. Its illustrated pathway bases on the formation of *line-defects*. The different realistic schemes of two opposed bilayers have been taken from Rappolt et al. [91]: starting from two membranes in close contact (E), a *line defect* spontaneously forms between them due to the spontaneous monolayer curvature (F). Here, the lipid molecules are allowed to adopt their intrinsic shape, i.e. to splay their chains and shorten accordingly. Next, the shortage of water content in this *line defect* is adjusted (G). From this point on, it is not difficult to imagine that a first inverted lipid tube pinches off (H). The last panel teaches us even more: around the first tube, six nearest-neighbor line defects are immediate (see *), and thus, further loci for further lipid tube formations are certain. Similar chain reactions have been describes also elsewhere [89], [92]. Laggner and Kriechbaum explained the high-cooperativity of this transition by a diffusion-free martensitic process [11], [93]. In their investigations, they found that the rapid temperature-jump experiments leads also to the appearance of water-poor intermediate L_α_ and H_2_ phases.

In addition, it should be pointed out that alternative linear precursors for the H_2_ phase have been proposed. First, monolayer-embedded lipid tubes may form via the coalescence of a “pearl-string” of inverted micelles [94]. This kind of “lipidic particle” fusion picture bases mainly on observations performed by freeze-fracture electron microscopy [41], [95]. Second, two opposed backbend monolayers were suggested to form rapidly as a result of the elongation of coalescent pairs of inverted micelle intermediates [89].

### Conclusions

In our present study, we carried out stopped-flow experiments combined with synchrotron SAXS for monitoring in-situ the structural transitions in DOPG/MO-based vesicles induced by rapidly added Ca^2+^ solutions. Our initial intention was to gain insight into the kinetics and the dynamics of the Ca^2+^-induced self-assembly, and to detect also the possible formation of intermediate phases. Under static conditions, it was reported that two different bicontinuous cubic phases (Im3m or Pn3m) form by the addition of Ca^2+^ ions to DOPG/MO-based ULVs and MLVs [1]. However revisiting the same studies but under rapid-mixing conditions, fast and unexpected bilayer to monolayer transitions were observed, i.e. already at low Ca^2+^ concentrations the L_α_ phase transformed within milliseconds into the H_2_ phase. Few seconds after the rapid mixing, no further changes in the fully formed H_2_ phase were detected. At 20°C, the transition from L_α_ to H_2_ phase occurs *via* an intermediate phase with a bilayer structure (possibly Pn3m phase). This intermediate structure has a short lifetime (100–400 ms). In contrast at 50°C, the impact of Ca^2+^ ions on the DOPG/MO membrane curvature is higher, and no formation of this intermediate phase was spotted. Our study demonstrates further that the sample preparation can have great influence on the calcium-induced nanostructures. For the formation of ‘pellets’, the excess of water has to be removed from the vesicular system, as done in ref. [1], and then under the influence of Ca^2+^ ions the formation of the cubic Im3m or Pn3m phases is observed (the published results have been reproduced). However, different results are obtained when *real* vesicles (lipidic nanostructures in excess water) are exposed to the same salt concentration. In this case, the H_2_ phase forms and it is stable for at least two weeks. The differences are most probably explained throughout lower Ca^2+^ affinity in the ‘pellet’ situation or alternatively by the unintentional removal of a fraction of DOPG molecules, which would increase the impact of MO lipids in the remaining pellet system. These findings also show how important it is to study self-assembled nanostructures under *realistic* excess of water conditions.

## Materials and Methods

### Materials

The lipids: monoolein (1-monooleoyl-rac-glycerol, MO, purity: 99%) was purchased from Sigma Chemical Co. (St. Louis, Missouri, USA), and 1,2-Dioleoyl-*sn*-Glycero-3-[Phospho-*rac*-(1-glycerol)] (Sodium Salt, purity: 99%) (DOPG) was obtained from Avanti Polar Lipids (Alabaster, AL, USA). Chloroform (CHCl_3_, purity: >99%) was supplied by Carl Roth GmbH (Karlsruhe, Germany). Calcium chloride dihydrate of analytical grade was supplied by Merck (Darmstadt, Germany). The used buffer was PIPES (pH 7.0). For rapid mixing, the stock salt solution containing 68 mM Ca^2+^ ions was prepared by dissolving the salt in the PIPES buffer. All ingredients were used without further purification.

### Preparation of DOPG/MO-based Vesicles

For the preparation of the vesicles, we followed a similar procedure to that reported by Awad et al. for forming MLVs [1]. In the absence of Ca^2+^ ions, the binary DOPG/MO mixture with molar ratio of 30∶70 was dispersed in the PIPES buffer. To follow the kinetics of the phase transitions in the rapid-mixing experiments, it was important to mix effectively the salt solution with the vesicles. This was achieved by carrying out the investigations with diluted DOPG/MO vesicles contain a total lipid weight of ∼7 wt%. Thus, the lipid concentration is different from which was used in [1] (30 wt% total lipid content). Another important difference is related to the preparation procedure of the samples for SAXS investigations. In our study, we investigated directly the formed vesicles coexisting with excess water without applying any additional protocols. In contrast, Awad et al. [1] applied an additional step of centrifuging the aqueous dispersions in order to remove excess water, and hence used the so-called ‘pellets’ of lipids for the SAXS measurements.

### Time-Resolved Synchrotron X-Ray Scattering Measurements

X-ray scattering patterns were recorded at the Austrian SAXS beamline (camera length 75 cm) at the synchrotron light source ELETTRA (Trieste, Italy) [96], [97] using a 1D position sensitive detector (Gabriel type), which covered the *q*-range (*q = *4π *sinθ/λ*, where *λ* is the wavelength and 2*θ* is the scattering angle) of interest from about 2π/300 to 2π/15 Å^−1^ at an X-ray energy of 8 keV. Silver behenate (CH_3_-(CH_2_)_20_-COOAg with a *d* spacing value of 58.38 Å) was used as a standard to calibrate the angular scale of the measured intensity [98]. Stop-flow mixing experiments on the lipid dispersions were performed with the commercial stopped-flow apparatus SFM-400 (Bio-logic Company, Claix, France) in combination with simultaneous time-resolved X-ray diffraction. The cell consists of four reservoirs (each vertical syringe of 10 ml volume is driven independently by stepping-motor) and three mixing champers. The geometry of this system allows evacuating easily air bubbles that could be formed during filling. For our study, two syringes have been operated and a total shot volume of 100 µl was chosen. When actuated by an electronic trigger signal, both the DOPG/MO-based vesicles and the solution of Ca^2+^ ions, respectively, were injected into the mixing chamber and finally pressed into an X-ray quartz capillary with a diameter of 1 mm (specified dead time 10 ms), which was thermostated as well as the syringes and the transfer lines of the stop-flow apparatus with a water bath (±0.1°C, Unistat CC, Huber, Offenburg, Germany). The temperature was set to 20, 25, and 50°C, respectively.

### X-ray data-analysis

In the time resolved X-ray scattering experiments, the first order *d* spacings of the L_α_ and inverse hexagonal H_2_ phase (reflections with the highest intensity) were derived from the SAXS diffraction pattern by standard procedures as described in [46]. All observed Bragg peaks were fitted by Lorentzian distributions after detector efficiency corrections and subtracting the background scattering from both water and the sample cell. The fittings were carried out with home-written procedures running under IDL 5.2 (Research Systems, Inc., USA). Few static scattering patterns were analyzed by the global analysis program (GAP) [99]. In this program, not only the lattice contributions [100], [101] are considered, but also a simple bilayer model is applied. This model uses one Gaussian for the headgroups and another for the hydrophobic core. A more detailed description of this model is given in Pabst's recent review [102].

## References

[1] Awad TS, Okamoto Y, Masum SMD, Yamazaki M (2005). Formation of cubic phases from large unilamellar vesicles of dioleoylphosphatidylglycerol/monoolein membranes induced by low concentrations of Ca^2+^.. Langmuir.

[2] Goodsell DS, Rosen MR (2004). Bionanotechnology Lessons from Nature.

[3] Zhang J, Wang ZL, Liu J, Chen S, Liu GY, Lockwood DJ (2002). Self-assembled nanostructures. Series title: Nanostructure Science and Technology.

[4] Couvreur P, Vauthier C (2006). Nanotechnology: intelligent design to treat complex disease.. Pharm Res.

[5] Boyd BJ (2005). Colloidal drug delivery.. Drug Deliv Rep.

[6] Zhao D, Huo Q, Feng J, Chmelka BF, Stucky GD (1998). Nonionic triblock and star diblock copolymer and oligomeric surfactant syntheses of highly ordered, hydrothermally stable, mesoporous silica structures.. J Am Chem Soc.

[7] Yaghmur A, Aserin A, Garti N (2002). Phase behavior of microemulsions based on food-grade nonionic surfactants: effect of polyols and short-chairs alcohols.. Colloids Surfaces A: Physicochem Eng Aspects.

[8] Weiss TM, Narayanan T, Wolf C, Gradzielski M, Panine P (2005). Dynamics of the self-assembly of unilamellar vesicles.. Phys Rev Lett.

[9] Gradzielski M (2004). Investigations of the dynamics of morphological transitions in amphiphilic systems.. Curr Opin Colloid Interface Sci.

[10] Gradzielski M, Grillo I, Narayanan T (2004). Dynamics of structural transitions in amphiphilic systems monitored by scattering techniques.. Progr Colloid Polym Sci.

[11] Kriechbaum M, Laggner P (1996). States of phase transitions in biological structures.. Progr Surf Sci.

[12] Laggner P, Amenitsch H, Kriechbaum M, Pabst G, Rappolt M (1998). Trapping of short-lived intermediates in phospholipid phase transitions: the L_α_*-Phase.. Faraday Discuss.

[13] Panine P, Finet S, Weiss T, Narayanan T (2006). Probing fast kinetics in complex fluids by combined rapid mixing and small-angle X-ray scattering.. Adv Colloid Interface Sci.

[14] Gradzielski M, Bergmeier M, Hoffmann H, Muller M, Grillo I (2000). Vesicle gel formed by a self-organization process.. J Phys Chem B.

[15] Moitzi C, Guillot S, Fritz G, Salentinig S, Glatter O (2007). Phase reorganization in self-assembled systems through interparticle material transfer.. Adv Mater.

[16] Grillo I, Kats EI, Muratov AR (2003). Formation and growth of anionic vesicles followed by small-angle neutron scattering.. Langmuir.

[17] Amenitsch H, Rappolt M, Teixeira CV, Majerowicz M, Laggner P (2004). In situ sensing of salinity in oriented lipid multilayers by surface X-ray scattering.. Langmuir.

[18] Bolze J, Pontoni D, Ballauff M, Narayanan T, Colfen H (2004). Time-resolved SAXS study of the effect of a double hydrophilic block-copolymer on the formation of CaCO_3_ from a supersaturated salt solution.. J Colloid Interface Sci.

[19] Allen SJ, Curran AR, Templer RH, Meijberg W, Booth PJ (2004). Folding kinetics of an α helical membrane protein in phospholipid bilayer vesicles.. J Mol Bio.

[20] Hu MZ-C, Zielke JT, Byers CH, Lin JS, Harris MT (2000). Probing the early-stage/rapid processes in hydrolysis and condensation of metal alkoxides.. J Mater Sci.

[21] Faraudo J, Travesset A (2007). Phosphatidic acid domains in membranes: effect of divalent counterions.. Biophys J.

[22] Hong K, Düzgünes N, Papahadjopoulos D (1982). Modulation of membrane fusion by calcium-binding proteins.. Biophys J.

[23] Papahadjopoulos D, Poste G (1975). Calcium-induced phase separation and fusion in phospholipid membranes.. Biophys J.

[24] Ortiz A, Killian JA, Verkleij AJ, Wilschut J (1999). Membrane fusion and the lamellar-to-inverted-hexagonal phase transition in cardiolipin vesicle systems induced by divalent cations.. Biophys J.

[25] Van Scyoc WS, Sorensen BR, Rusinova E, Laws WR, Ross JBA (2002). Calcium binding to calmodulin mutants monitored by domain-specific intrinsic phenylalanine and tyrosine fluorescence.. Biophys J.

[26] Crossthwaite AJ, Seebacher T, Masada N, Ciruela A, Dufraux K (2005). The cytosolic domains of Ca^2+^-sensitive adenylyl cyclases dictate their targeting to plasma membrane lipid rafts.. J Biol Chem.

[27] Szule JA, Jarvis SE, Hibbert JE, Spafford JD, Braun JEA (2003). Calcium-triggered membrane fusion proceeds independently of specific presynaptic proteins.. J Biol Chem.

[28] Chasserot-Golaz S, Vitale N, Umbrecht-Jenck E, Knight D, Gerke V (2005). Annexin 2 promotes the formation of lipid microdomains required for calcium-regulated exocytosis of dense-core vesicles.. Mol Biol Cell.

[29] Vest RS, Gonzales LJ, Permann SA, Spencer E, Hansen LD (2004). Divalent cations increase lipid order in erythrocytes and susceptibility to secretory phospholipase A_2_.. Biophys J.

[30] Kharakoz D, Tien HT, Ottova-Leitmannova A (2003). Chain-ordering phase transition in bilayer: kinetic mechanism and its physicochemical and physiological implications.. Planar lipid bilayers (BLMs) and their applications. Membrane Science and technology series, 7.

[31] Krebs J, Meyers RA (1995). Calcium Biochemistry.. Encyclopedia of molecular biology and molecular medicine. Vol. 1.

[32] Garidel P, Blume A, Hübner WA (2000). Fourier transform infrared spectroscopic study of the interaction of alkaline earth cations with the negatively charged phospholipid 1,2-dimyristoyl-sn-glycero-3-phosphoglycerol.. Biochim Biophys Acta.

[33] Garidel P, Blume A (2000). Calcium induced nonideal mixing in liquid-crystalline phosphatidylcholine-phosphatidic acid bilayer membranes.. Langmuir.

[34] Sinn CG, Antonietti M, Dimora R (2006). Binding of calcium to phosphatidylcholine-phosphatidylserine membranes.. Colloids Surfaces A: Physicochem Eng Aspects.

[35] De Kruijff B, Killian JA, Rietveld AG, Kusters R, Epand RM (1997). Phospholipid structure and *Escherichia* Coli Membranes.. Lipid polymorphism and membrane properties. Current topics in membrane.

[36] Li SJ, Yamashita Y, Yamazaki M (2001). Effect of electrostatic interactions on phase stability of cubic phases of membranes of monoolein/dioleoylphosphatidic acid mixtures.. Biophys J.

[37] Verkleij AJ, de Maagd R, Leunissen-Bijvelt J, de Kruijff B (1982). Divalent cations and chlorpromazine can induce non-bilayer structures in phosphatidic acid-containing model membranes.. Biochim Biophys Acta.

[38] De Kruijff B, Verkleij AJ, Leunissen-Bijvelt J, Van Echteld CJA, Hille J (1982). Further aspects of the Ca^2+^-dependent polymorphism of bovine heart cardiolipin.. Biochim Biophys Acta.

[39] Killian JA, Koorengevel MC, Bouwstra JA, Gooris G, Dowhan W (1994). Effect of divalent cations on lipid organization of cardiolipin isolated from Escherichia coli strain AH930.. Biochim Biophys Acta.

[40] Ellens H, Siegel DP, Alford D, Yeagle PL, Boni L (1989). Membrane fusion and inverted phases.. Biochemistry.

[41] Colotto A, Epand RM (1997). Structural study of the relationship between the rate of membrane fusion and the ability of the fusion peptide of influenza virus to perturb bilayers.. Biochemistry.

[42] Epand RM, Epand RM (1997). Modulation of lipid polymorphism by peptides.. Lipid polymorphism and membrane properties. Current topics in membrane.

[43] Siegel DP (1999). The modified stalk mechanism of lamellar/inverted phase transitions and its implications for membrane fusion.. Biophys J.

[44] Yaghmur A, Laggner P, Zhang S, Rappolt M (2007). Tuning curvature and stability of monoolein bilayers by short surfactant-like designer peptides.. PLoS ONE.

[45] Luzzati V (1997). Biological significance of lipid polymorphism: the cubic phases.. Curr Opin Struct Biol.

[46] Lindblom G, Rilfors L (1989). Cubic phases and isotropic structures formed by membrane lipids - possible biological relevance.. Biochim Biophys Acta.

[47] De Kruijff B (1997). Lipids beyond the bilayer.. Nature.

[48] Rappolt M, Leitmannova A (2007). The biologically relevant lipid mesophases as “seen” by X-rays.. Planar lipid bilayers and liposomes.

[49] Simidjiev I, Stoylova S, Amenitsch H, Jávorfi T, Mustárdy L (2000). Self-assembly of large, ordered lamellae from non-bilayer lipids and integral membrane proteins *in vitro*.. PNAS.

[50] Almsherqi ZA, Kohlwein SD, Deng Y (2006). Cubic membranes: a legend beyond the flatland of cell membrane organization.. J Cell Biol.

[51] Hyde S, Andersson S, Larsson K, Blum Z, Landh T (1997). The language of shape. The role of curvature in condensed matter: physics, chemistry, biology, Elsevier book series.

[52] Patton JS, Carey MC (1979). Watching fat digestion.. Science.

[53] Yaghmur A, de Campo L, Sagalowicz L, Leser ME, Glatter O (2006). Control of the internal structure of MLO-based isasomes by the addition of diglycerol monooleate and soybean phosphatidylcholine.. Langmuir.

[54] Larsson K (1983). Two cubic phases in monoolein/water system.. Nature.

[55] Lutton ES (1965). Phase behavior of aqueous systems of monoglycerides.. JAOCS.

[56] Qiu H, Caffrey M (2000). The phase diagram of the monoolein/water system: metastability and equilibrium aspects.. Biomaterials.

[57] Nilsson A, Holmgren A, Lindblom G (1994). An FTIR study of the hydration and molecular ordering at phase transitions in the monooleoylglycerol/water system.. Chem Phys Lipids.

[58] Chernik GG (2000). Phase studies of surfactant-water systems.. Curr Opin Colloid Interface Sci.

[59] Rappolt M, Di Gregorio GM, Almgren M, Amenitsch H, Pabst G (2006). Non-equilibrium formation of the cubic Pn3m phase in a monoolein/water system.. Europhy Lett.

[60] Andersson A-S, Rilfors L, Orädd G, Lindblom G (1998). Total lipids with short and long acyl chains from *Acholeplasma* form nonlamellar phases.. Biophys J.

[61] Larsson K (2000). Aqueous dispersions of cubic lipid-water phases.. Curr Opin Colloid Interface Sci.

[62] Gustafsson J, Ljusberg-Wahren H, Almgren M, Larsson K (1996). Cubic lipid-water phase dispersed into submicron particles.. Langmuir.

[63] De Campo L, Yaghmur A, Sagalowicz L, Leser ME, Watzke H (2004). Reversible phase transitions in emulsified nanostructured lipid systems.. Langmuir.

[64] Spicer PT, Schwarz JA, Contescu C, Putyera K (2004). Cubosomes: bicontinuous liquid crystalline nanoparticles.. Dekker encyclopedia of nanoscience and nanotechnology.

[65] Yaghmur A, de Campo L, Sagalowicz L, Leser ME, Glatter O (2005). Emulsified microemulsions and oil-containing liquid crystalline phases.. Langmuir.

[66] Angelova A, Angelov B, Papahadjopoulos-Sternberg B, Bourgaux C, Couvreur P (2005). Protein driven patterning of self-assembled cubosomic nanostructures: long oriented nanoridges.. J Phys Chem B.

[67] Yaghmur A, de Campo L, Salentinig S, Sagalowicz L, Leser ME (2006). Oil-loaded monolinolein-based particles with confined inverse discontinuous cubic structure (*Fd*3*m*).. Langmuir.

[68] Pabst G, Koschuch R, Pozo-Navas B, Rappolt M, Lohner K (2003). Structural analysis of weakly ordered membrane stacks.. J Appl Crystallogr.

[69] Garidel P, Blume A (2000). Thermodynamic characterization of bile salt aggregation as a function of temperature and ionic strength using isothermal titration calorimetry.. Langmuir.

[70] Pederson UR, Leidy C, Westh P, Peters GH (2006). The effect of calcium on the properties of charged phospholipid bilayers.. Biochim Biophys Acta.

[71] Pabst G, Hodzic A, Strancar J, Danner S, Rappolt M (2007). Rigidification of neutral lipid bilayers in the presence of salts.. Biophys J.

[72] Lehrmann R, Seelig J (1994). Adsorption of Ca^2+^ and La^3+^ to bilayer membranes: measurement of the adsorption enthalpy and binding constant with titration calorimetry.. Biochim Biophys Acta.

[73] Böckmann RA, Grubmüller H (2004). Multistep binding of divalent cations to phospholipid bilayers: A molecular dynamics study.. Angew Chem Int Ed Engl.

[74] Rappolt M, Pressl K, Pabst G, Laggner P (1998). L_α_-phase separation in phosphatidylcholine-water systems induced by alkali chlorides.. Biochim Biophys Acta.

[75] Rappolt M, Pabst G, Amenitsch H, Laggner P (2001). Salt-induced phase separation in the liquid crystalline phase of phosphatidylcholines.. Colloids Surfaces A: Physicochem Eng Aspects.

[76] Isrealachvilli JN, Mitchell DJ, Ninham BW (1976). Theory of self-assembly of hydrocarbon amphiphiles into micelles and bilayers.. J Chem Soc Faraday Trans II.

[77] Masum SMD, Li SJ, Tamba Y, Yamashita Y, Tanaka T (2003). Effect of de Novo designed peptides interacting with the lipid-membrane interface on the stability of the cubic phases of the monoolein membrane.. Langmuir.

[78] Kamo T, Nakano M, Leesajakul W, Sugita A, Matsuoka H (2003). Nonlamellar liquid crystalline phases and their particle formation in the egg yolk phosphatidylcholine/diolein system.. Langmuir.

[79] Nakano M, Kamo T, Sugita A, Handa T (2005). Detection of bilayer packing stress and its release in lamellar-cubic phase transition by time-resolved fluorescence anisotropy.. J Phys Chem B.

[80] Pitzalis P, Monduzzi M, Krog N, Larsson H, Ljusberg-Wahren H (2000). Characterization of the liquid-crystalline phases in the glycerol monooleate/diglycerol monooleate/water system.. Langmuir.

[81] Garidel P, Blume A (1999). Interaction of alkaline earth cations with the negatively charged phospholipid 1,2-dimyristoyl-*sn*-glycero-3-phosphoglycerol: a differential scanning and isothermal titration calorimetric study.. Langmuir.

[82] Boggs JM, Rangaraj G (1983). Investigations of the metastable phase behavior of phosphatidylglycerol with divalent cations by calorimetry and manganese ion binding measurements.. Biochemistry.

[83] Garidel P, Forster G, Richter W, Kunst BH, Rapp G (2000). 1,2-Dimyristoyl-sn-glycero-3-phosphoglycerol (DMPG) divalent cation complexes: an X-ray scattering and freeze-fracture electron microscopy study.. Phys Chem Chem Phys.

[84] Arseneault M, Lafleur M (2006). Isothermal titration calorimetric study of calcium association to lipid bilayers: influence of the vesicle preparation and composition.. Chem Phys Lipids.

[85] Roux M, Bloom M (1990). Ca^2+^, Mg^2+^, Li^+^, Na^+^, and K^+^ distributions in the headgroup region of binary membranes of phosphatidylcholine and phosphatidylserine as seen by deuterium NMR.. Biochemistry.

[86] Briggs J, Chung H, Caffrey M (1996). The temperature-composition phase diagram and mesophase structure characterization of the monoolein/water system.. J Phys II France.

[87] Shearman GC, Ces O, Templer RH, Seddon JM (2006). Inverse lyotropic phases of lipids and membrane curvature.. J Phys Condens Matter.

[88] Hui SW, Stewart TP, Boni LT, Yeagle PL (1981). Membrane fusion through point defects in bilayers.. Science.

[89] Siegel DP (1986). Inverted micellar intermediates and the transitions between lamellar, cubic, and inverted hexagonal lipid phases.. Biophys J.

[90] Seddon JM (1990). Structure of the inverted hexagonal (H_II_) phase, and non-lamellar phase transitions of lipids.. Biochim Biophys Acta.

[91] Rappolt M, Hodzic A, Sartori B, Ollivon M, Laggner P (2008). Conformational and hydrational properties during the L_β_ to L_α_ and L_α_ to H_II_ phase transition in phosphatidylethanolamine.. Chem Phys Lipids.

[92] Caffrey M (1985). Kinetics and mechanism of the lamellar gel/liquid crystal and lamellar/inverted hexagonal phase transition in phoshatidylethanolamine: a real-time X-ray diffraction study using synchrotron radiation.. Biochemistry.

[93] Laggner P, Kriechbaum M (1991). Phospholipid phase transitions: kinetics and structural mechanisms.. Chem Phys Lipids.

[94] Hui SW, Stewart TP, Boni LT (1983). The nature of lipidic particles and their roles in polymorphic transitions.. Chem Phys Lipids.

[95] Verkleij AJ, van Echteld CJA, Gerritsen WJ, Cullis PR, de Kruijff B (1980). The lipidic particle as an intermediate structure in the membrane fusion and bilayer to hexagonal (H_ii_) transitions.. Biochim Biophys Acta.

[96] Amenitsch H, Rappolt M, Kriechbaum M, Mio H, Laggner P (1998). First performance assessment of the small-angle X-ray scattering beamline at ELETTRA.. J Synchrotron Rad.

[97] Bernstorff S, Amenitsch H, Laggner P (1998). High-throughput asymmetric double-crystal monochromator of the SAXS beamline at ELETTRA.. J Synchrotron Rad.

[98] Huang TC, Toraya H, Blanton TN, Wu Y (1993). X-ray powder diffraction analysis of silver behenate, a possible low-angle diffraction standard.. J Appl Crystallogr.

[99] Pabst G, Rappolt M, Amenitsch H, Laggner P (2000). Structural information from multilamellar liposomes at full hydration: full q-range fitting with high quality x-ray data.. Phys Rev E.

[100] Caillé A (1972). Remarques sur la diffusion des rayons X dans les smectiques.. A C R Acad Sc Paris B.

[101] Zhang R, Tristram-Nagle S, Sun W, Headrick RL, Irving TC (1996). Small-angle x-ray scattering from lipid bilayers is well described by modified Caillé theory but not by paracrystalline theory.. Biophys J.

[102] Pabst G (2006). Global properties of biomimetic membranes: perspectives on molecular features.. Biophys Rev Letters.

